# Association of neutrophil–lymphocyte ratio with survival in peripheral early-stage non-small cell lung cancer after stereotactic body radiation therapy

**DOI:** 10.1186/s12885-023-10719-3

**Published:** 2023-03-18

**Authors:** Karen Huang, Sharan Prasad, Sung Jun Ma, Han Yu, Austin J. Iovoli, Mark K. Farrugia, Elizabeth U. Dexter, Todd L. Demmy, Nadia K. Malik, Anurag K. Singh

**Affiliations:** 1 Department of Radiation Medicine, Roswell Park Comprehensive Cancer Center, 665 Elm Street, Buffalo, NY 14203 USA; 2 Cornell University, 410 Thurston Avenue, Ithaca, NY 14850 USA; 3 Department of Biostatistics and Bioinformatics, Elm and Carlton Streets, Buffalo, NY 14263 USA; 4 Department of Thoracic Surgery, Elm and Carlton Streets, Buffalo, NY 14263 USA

**Keywords:** Non-small cell lung cancer, Neutrophil–lymphocyte ratio, Stereotactic body radiation therapy, Survival

## Abstract

**Background:**

The role of neutrophil–lymphocyte ratio (NLR) as a predictor for survival in single fraction SBRT-treated non-small cell lung cancer (NSCLC) patients remains unclear. We performed an observational cohort study to determine the role of pretreatment NLR in predicting survival of early-stage NSCLC patients after single fraction SBRT.

**Methods:**

A single-institution database of peripheral early-stage NSCLC patients treated with SBRT from February 2007 to May 2022 was queried. Optimal threshold of neutrophil–lymphocyte ratio (NLR) was defined based on maximally selected rank statistics. Cox multivariable analysis (MVA), Kaplan–Meier, and propensity score matching were performed to evaluate outcomes.

**Results:**

A total of 286 patients were included for analysis with median follow up of 19.7 months. On Cox multivariate analysis, as a continuous variable, NLR was shown to be an independent predictor of OS (adjusted hazards ratio [aHR] 1.06, 95% CI 1.02–1.10, *p* = 0.005) and PFS (aHR 1.05, 95% CI 1.01–1.09, *p* = 0.013). In addition, NLR was associated with DF (aHR 1.11, 95% CI 1.05–1.18, *p* < 0.001). Maximally selected rank statistics determined 3.28 as the cutoff point of high NLR versus low NLR. These findings were confirmed upon propensity matching.

**Conclusions:**

Pretreatment NLR is an independent predictor for survival outcomes of peripheral early-stage NSCLC patients after single fraction SBRT.

**Supplementary Information:**

The online version contains supplementary material available at 10.1186/s12885-023-10719-3.

## Introduction

Lung cancer is the leading cause of cancer-related deaths in the United States, with 235,760 new cases and 131,880 deaths in 2021 [1]. Standard of care for patients with early-stage non-small cell lung cancer (NSCLC) is surgical resection [2]. For patients medically inoperable, stereotactic body radiation therapy (SBRT) is utilized as definitive treatment [3]. Various lung SBRT dose fractionation regimens are employed globally with single-fraction SBRT found to be equally effective as multi-fraction regimens [4–11].

For several malignancies, neutrophil–lymphocyte ratio (NLR) has been associated with survival outcomes of patients treated with SBRT [12–14]. In NSCLC, previous studies have demonstrated that NLR predicts overall survival (OS) [15–20], and one study linked NLR and local recurrence [21]. No study previously reported on NLR and single fraction SBRT. To investigate the correlation of NLR to outcomes, we performed a single-institution, observational cohort study involving patients with peripheral early-stage NSCLC who underwent predominantly single-fraction SBRT.

## Materials and Methods

Our cohort study was approved by the Roswell Park Comprehensive Cancer Center Institutional Review Board (EDR 171,710). It follows the Strengthening the Reporting of Observations Studies in Epidemiology (STROBE) reporting guideline.

The cohort database was selected from NSCLC patients treated with SBRT at Roswell Park Comprehensive Cancer Center between February 2007 to May 2022. Consecutive patients with peripheral early-stage NSCLC (T1-2N0M0) and a complete blood count within six months of the start of SBRT treatment were included. Patients were excluded from the analysis if they had missing NLR data. Patients treated with SBRT regimens of more than 3 fractions were excluded as these regimens (i.e., 5 fractions) were reserved for patients with centrally located lesions with higher risk of toxicity which could affect survival [22–25].

Other clinically relevant variables such as age, gender, race, Karnofsky Performance Status (KPS), histology (adenocarcinoma, squamous cell carcinoma, NSCLC not otherwise specified), primary cancer site, T-stage, radiation fractions, smoking status, year of treatment, and tumor location were obtained from the electronic health record (EHR). All missing values were coded as unknown. Patient race was separated as White, African American, American Indian/Alaska Native, Asian, Hispanic, and unknown or declined to answer. Non-white patients were grouped together as a single category because of the small subgroup sample sizes.

Primary outcomes were overall survival (OS) and progression-free survival (PFS). OS was determined from the time interval encompassing the start of treatment to the last known follow-up or death (from any cause). PFS was determined from the time of the start of the treatment to any tumor recurrence, the last known follow up, or death. The secondary outcomes were local failure (LF), nodal failure (NF), and distant failure (DF). Secondary outcomes were determined from the time between the start of treatment to a failure at same cancer site, thoracic nodal station, or extra thoracic or contralateral lung failure, respectively. All tumor recurrences were determined through multidisciplinary discussion based on radiographic findings and, when available, biopsy results of metastatic sites. For patients with multiple failure events either synchronously or metachronously during their follow up period, all failure events were counted separately for analysis.

### Statistical analysis

To visualize the relationship between patient survival and pre-treatment NLR as a continuous variable, a nonlinear Cox regression model with restricted cubic splines (RCS) was performed as previously shown [26]. RCS is a smooth, piecewise polynomial function that visualizes the association between a variable and an outcome without any prior assumption in the association. The model was constructed for OS and PFS using 3 knots at the 10^th^, 50^th^, and 90^th^percentiles based on the lowest Akaike information criterion [27].

Cox multivariable analysis (MVA) was used to investigate the prognostic role of pre-treatment NLR as a continuous variable in early-stage peripheral NSCLC patients, with the addition of clinically relevant variables (age, gender, race, KPS, histology, site of cancer, T-stage, fractions of radiation, smoking status, year of radiation treatment, and tumor location). Furthermore, Fine-Gray competing risk MVA was also performed to evaluate secondary outcomes (LF, NF, and DF). Kaplan–Meier method and log-rank tests were used to examine the univariate association between OS and PFS with pre-treatment NLR after dichotomization. An optimal cutoff for high versus low NLR was obtained by using an outcome-based process by maximizing the log-rank test statistic and survival differences as previously described [26, 28, 29]. The cutoff was searched between the NLR quantiles of 0.1 and 0.9. The optimal cutoff was analyzed for both OS and PFS, and patients were then stratified into two cohorts, high versus low pre-treatment NLR, by above versus below the optimal cutoff. Based on optimal cutoff, 1- and 3-year survival and tumor controls were calculated for analysis. Note that the searching of optimal cutoff by the log-rank statistic is conditional on the overall significant association between NLR and the survival outcomes. Therefore, multiple testing during the cutoff searching is not an issue.

To limit selection bias, propensity score matching was performed using the optimal cutoff value calculated for NLR. The two cohorts, high and low NLR, were matched based on the previous variables listed above. Matching was based on nearest neighbor method in a 1:1 ratio with no replacement using a caliper distance of 0.2 [30]. Furthermore, Cox and Fine-Gray regression models were performed to evaluate OS, PFS, and secondary outcomes after matching. Logistic regression was performed to identify any related variables to high versus low NLR. A subgroup analysis was performed among patients treated with single-fraction SBRT to see whether our findings would be consistent.

All tests were two-sided and p values less than 0.05 were considered statistically significant. Adjusted hazard ratios (aHR) and 95% confidence intervals (CI) were reported for analysis. Data analyses were done using R (version 4.1.2, R Project for Statistical Computing, Vienna, Austria).

## Results

A total of 286 patients (164 female [57.3%]; median [IQR] age 76 [69–81] years) were included in our analysis (Table 1). Most patients had adenocarcinoma (160, 55.9%) or squamous cell carcinoma (93, 32.5%). The majority of tumors were clinical stage T1 (235, 82.2%). SBRT prescriptions with heterogeneity correction were 27 Gy in 1 fraction (211, 72.8%) and 54 Gy in 3 fractions (75, 26.2%). The median NLR was 3.06 ([IQR] 2.21–4.33). There were 15 local failures (5.2%), 27 nodal failures (9.4%), and 50 distant failures (17.5%). The median follow up was 19.7 months ([IQR] 9.78–35.48).

**Table 1:** Baseline characteristics

	Patients, No. (%)		
	All (*n* = 286)	Low NLR (*n* = 158)	High NLR (*n* = 128)
Age			
< 65	48 (16.8)	27 (17.1)	21 (16.4)
≥ 65	238 (83.2)	131 (82.9)	107 (83.6)
Gender			
Male	122 (42.7)	61 (38.6)	61 (47.7)
Female	164 (57.3)	97 (61.4)	67 (52.3)
Race			
White	271 (94.8)	148 (93.7)	123 (96.1)
Other	15 (5.2)	10 (6.3)	5 (3.9)
KPS			
70–100	178 (62.2)	101 (63.9)	77 (60.2)
< 70	108 (37.8)	57 (36.1)	51 (39.8)
Histology			
Adenocarcinoma	160 (55.9)	92 (58.2)	68 (53.1)
Squamous Cell	93 (32.5)	47 (29.7)	46 (35.9)
NSCLC (NOS)	33 (11.5)	19 (12.0)	14 (10.9)
Site			
Left	140 (49.0)	80 (50.6)	60 (46.9)
Right	146 (51.0)	78 (49.4)	68 (53.1)
T staging			
1	235 (82.2)	136 (86.1)	99 (77.3)
2	51 (17.8)	22 (13.9)	29 (22.7)
Fractions			
1	211 (72.8)	117 (74.1)	94 (73.4)
3	75 (26.2)	41 (25.9)	34 (26.6)
Smoking Status			
Current	79 (27.6)	50 (31.6)	29 (22.7)
Former	188 (65.7)	97 (61.4)	91 (71.1)
Never	19 (6.6)	11 (7.0)	8 (6.3)
Year of radiation			
2013 or earlier	220 (76.9)	125 (79.1)	95 (74.2)
2013 or later	66 (23.1)	33 (20.9)	33 (25.8)

*N* number, *KPS* Karnofsky performance, *NOS* not otherwise specified

**Fig. 1:**
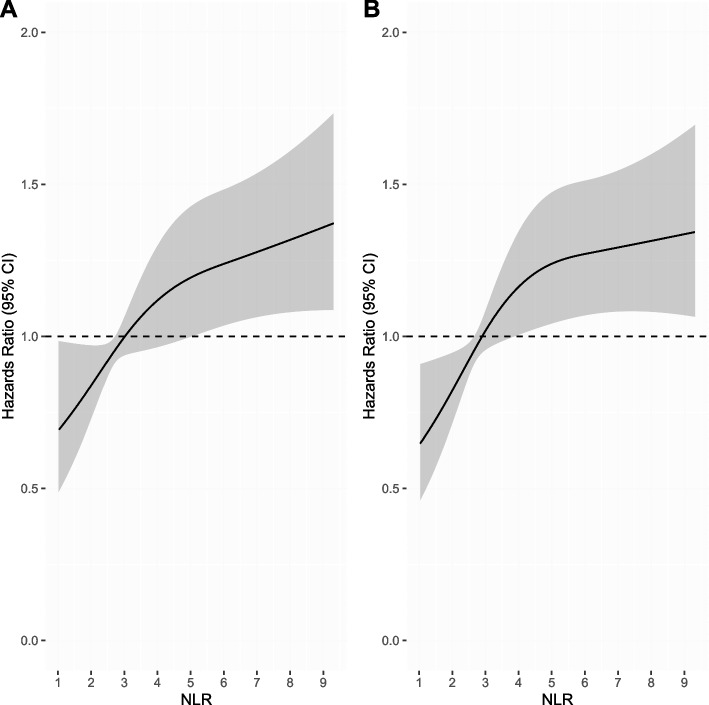
Nonlinear Cox regression for overall (**A**) and progression-free (**B**) survival based on neutrophil–lymphocyte ratio (NLR) as a continuous variable

The nonlinear Cox regression model showed worsening OS and PFS without plateau in a continuous fashion as NLR increased (Fig. 1). On Cox MVA, as a continuous variable, elevated NLR was associated with poorer OS (adjusted hazards ratio [aHR] 1.06, 95% CI 1.02–1.10, *p* = 0.005) and PFS (aHR 1.05, 95% 1.01–1.09, *p* = 0.01; Table 2). In addition, age and KPS showed an expected association with OS, and KPS also showed an expected association with PFS (Table 2). Fine-Gray competing risk MVA indicated that elevated NLR was significantly related to increased likelihood of DF (aHR 1.11, 95% CI 1.05–1.18, *p* < 0.001), but not related to NF (aHR 1.08, 95% CI 0.97–1.21, *p* = 0.16; eTable 1). The number of LF events were too few to evaluate by MVA.

**Table 2:** Cox multivariate analysis for overall and progression-free survival outcomes

	Overall Survival		Progression-Free Survival	
	HR (95% CI)	*P* value	HR (95% CI)	*P* value
NLR	1.06 (1.02–1.10)	0.005^a^	1.05 (1.01–1.09)	0.01^a^
Age				
< 65	1 [Reference]	NA	1 [Reference]	NA
≥ 65	2.56 (1.47–4.46)	< 0.001^a^	1.47 (0.92–2.36)	0.11
Gender				
Male	1 [Reference]	NA	1 [Reference]	NA
Female	0.91 (0.66–1.26)	0.57	0.83 (0.60–1.13)	0.24
Race				
White	1 [Reference]	NA	1 [Reference]	NA
Other	1.22 (0.59–2.51)	0.6	1.26 (0.62–2.56)	0.52
KPS				
70–100	1 [Reference]	NA	1 [Reference]	NA
< 70	1.59 (1.12–2.24)	0.009^a^	1.43 (1.03–1.99)	0.03^a^
Histology				
Adenocarcinoma	1 [Reference]	NA	1 [Reference]	NA
Squamous Cell	1.16 (0.81–1.65)	0.42	1.11 (0.80–1.56)	0.53
NSCLC (NOS)	1.21 (0.72–2.02)	0.47	0.93 (0.55–1.55)	0.77
Site				
Left	1 [Reference]	NA	1 [Reference]	NA
Right	0.93 (0.66–1.30)	0.66	0.87 (0.63–1.21)	0.41
T staging				
1	1 [Reference]	NA	1 [Reference]	NA
2	1.45 (0.99–2.12)	0.06	1.39 (0.96–2.01)	0.09
Fractions				
1	1 [Reference]	NA	1 [Reference]	NA
3	1.37 (0.97–1.94)	0.07	1.34 (0.97–1.87)	0.08
Smoking Status				
Current	1 [Reference]	NA	1 [Reference]	NA
Former	0.80 (0.54–1.17)	0.24	0.75 (0.52–1.07)	0.11
Never	0.80 (0.35–1.82)	0.6	0.67 (0.30–1.50)	0.32
Year of radiation				
2013 or earlier	1 [Reference]	NA	1 [Reference]	NA
2013 or later	0.76 (0.54–1.07)	0.12	0.84 (0.60–1.17)	0.3

*aHR* adjusted hazards ratio, *CI* confidence interval, *KPS* Karnofsky performance, *NOS* not otherwise specified

^a^statistically significant

**Fig. 2:**
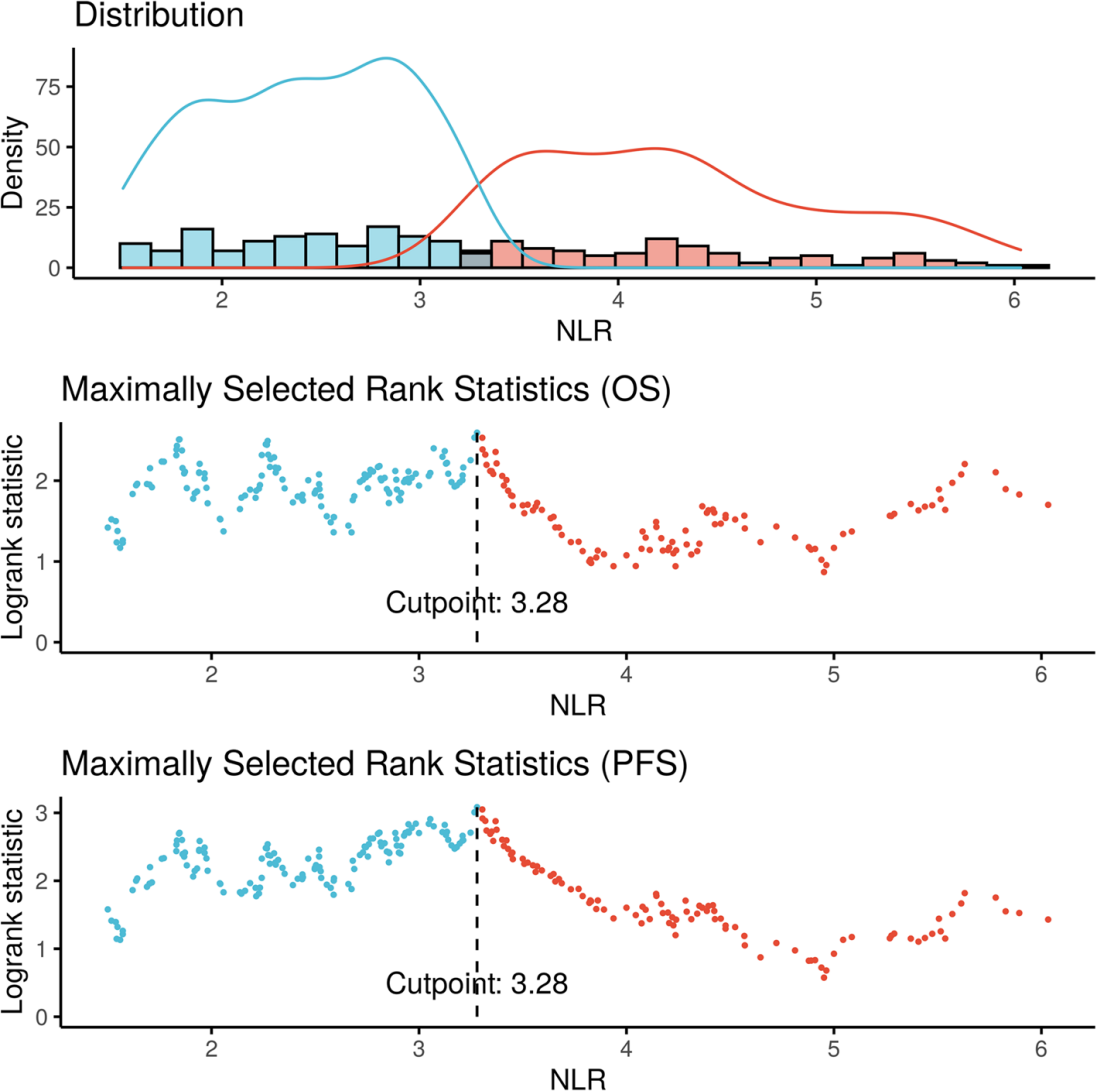
Distribution of neutrophil–lymphocyte ratio (NLR) and threshold assessment using maximum log-rank test statistic

**Fig. 3:**
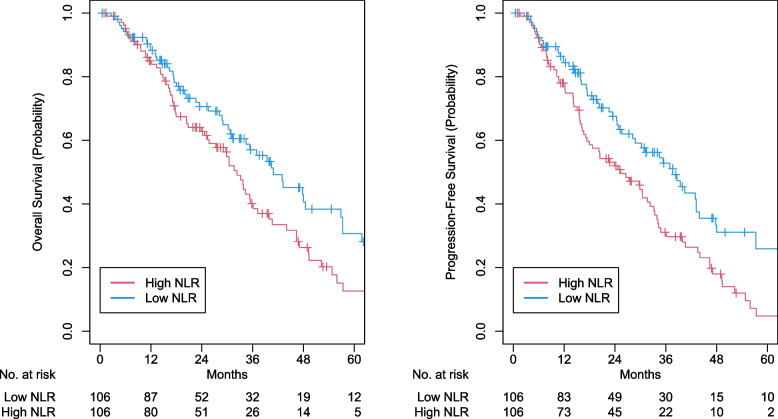
Kaplan–Meier curves for high versus low neutrophil–lymphocyte ratio (NLR) for overall survival (OS) and progression-free survival (PFS) after propensity score matching

**Fig. 4:**
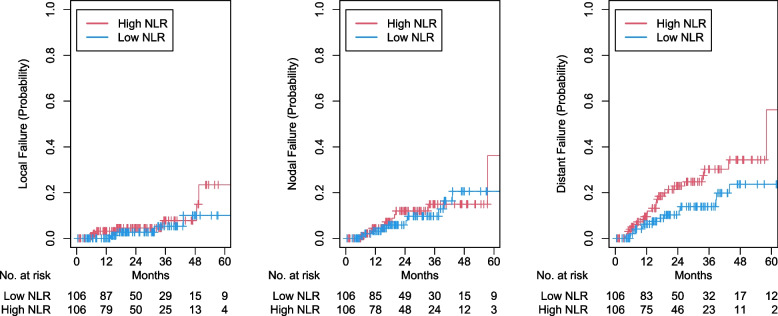
Cumulative incidence curves for high versus low neutrophil–lymphocyte ratio (NLR) for local failure, nodal failure, and distant failure after propensity score matching

Determined by maximally selected rank statistics, the optimal cutoff value of NLR was 3.28 (Fig. 2). There were 131 patients and 161 patients in the high (≥ 3.28) and low NLR groups, respectively. After propensity matching, high NLR was associated with worse OS (2-year OS 62.9% vs 70.6%; aHR 1.50, 95% CI 1.05–2.15, *p* = 0.027), and PFS (2-year PFS 52.0% vs 67.6%; aHR 1.68, 95% CI 1.19–2.38, *p* = 0.003), and DF (2-year DF 22.9% vs 10.3%; aHR 1.97, 95% CI 1.01–3.83, *p* = 0.045; eTable 2). However, high NLR was not associated with NF (2-year NF 12.0% vs 5.8%; aHR 1.22, 95% CI 0.52–2.87, *p* = 0.65) and LF (2-year LF 4.5% vs 2.6%; aHR 1.57, 95% CI 0.572–0.792, *p* = 0.43). Kaplan- Meier curves were generated for OS, PFS, LF, NF, and DF for high versus low NLR (Fig. 3 and 4). In logistic regression, there were no statistically significant variables related to NLR (eTable3). Comparisons between our survival outcomes and those in other published studies analyzing NLR in patients treated with SBRT are described in Table 3.

**Table 3:** Characteristics of studies on the role of pre-treatment NLR on NSCLC patients treated with SBRT

Author	Year	Region	# pts	Median OS (months)	3- year OS (%)		Median NLR (range)	NLR cutoff	OS		Tumor recurrence		
					High NLR	Low NLR			HR (95% CI)	*P* value	HR (95% CI)	*P* value	Type
Current	2022	USA	286	23.48	40.1	57.1	3.06 (2.21–4.33)	3.28	1.06 (1.02–1.10)	0.005^a^	1.08 (0.97–1.21)	0.16	nodal
											1.11(1.05–1.18)	< 0.001^a^	distant
AduQuaye	2022	Canada	61	36	NR	NR	3.42 (0.27–13.69)	NR	1.26 (1.04–1.53)	0.017^a^	1.05(1–1.1)	0.021^a^	local
Kotha	2021	USA	389	31.5	NR	NR	3.0 (0.4–42)	4	1.44 (1.12–1.86)	0.01^a^	NR	NR	NR
Sebastian	2019	USA	156	32.9	NR	NR	3.6 (0.2–41.8)	3.6	1.91 (1.09–3.33)	0.023^a^	1.21 (0.42–3.49)	0.73	local
											1.30 (0.61–2.74)	0.5	regional nodal
											1.95 (0.81–4.72)	0.14	distant
Luo	2018	China	63	NR	NR	NR	2.47 (0.86–7.29)	2.06	1.489 (1.096–2.021)^b^	0.011^b^	NR	NR	NR
Shaverdian	2016	USA	118	NR	61.0	92.0	2.79(0.95–11.6)	2.18	1.477 (NR)	0.008^a^	0.816 (NR)	0.456	locoregional
											1.255 (NR)	0.438	distant
Giuliani	2016	Canada	122	43.7	41.2	66.2	3.0 (0.3–22.0)	3	1.22 (1.08–1.38)	0.001^a^	NR	NR	NR
Cannon	2015	USA	59	43	NR	NR	2.8 (0.5–33.0)	2.98	NR	0.005^a^	NR	0.937	nonlocal

*pts* patients, *NR* not reported

^a^statistically significant; ^b^calculated with univariate analysis (instead of multivariate)

Among those treated with single-fraction SBRT (n = 211) on Cox MVA, NLR as a continuous variable remained statistically significant for OS (aHR 1.09, 95% CI 1.03–1.15, *p* = 0.001) and PFS (aHR 1.08, 95% CI 1.03–1.14, *p* = 0.002). On Fine-Gray competing risk MVA, NLR was not statistically significant for nodal failure (aHR 1.06, 95% CI 0.91–1.25, *p* = 0.4). Number of local (n = 10) and distant failures (n = 27) were too few to analyze in MVA.

## Discussion

Pre-treatment NLR was significantly associated with distant failure, progression-free, and overall survival. This is the first study to show that pre-treatment NLR was a statistically significant predictor of increased distant failure and poor PFS in early-stage NSCLC patients treated with single fraction SBRT.

Neutrophils can either stimulate or suppress the cytotoxic T-cell response. The balance between stimulation and suppression may be related to the ratio of neutrophils to lymphocytes [31]. As a biomarker of systemic inflammation, NLR has been shown to serve a prognostic role in various cancers [12–14]. In NSCLC patients treated with either surgical or non-surgical methods, NLR has been shown to be a prognostic factor [32, 33].

Our findings are consistent with previous studies demonstrating high NLR was associated with worse overall survival outcomes in NSCLC patients treated with SBRT [15–21]. On Cox multivariate analysis, high NLR was continuously associated with poor OS and PFS. After propensity matching in our study, patients with a NLR greater than 3.28 were also significantly more likely to have an inferior OS and PFS outcomes after radiation therapy. Our optimal cutoff value of 3.28 was similar to prior studies (range: 2.06–4.00) that analyzed the role of NLR on prognosis of early-stage NSCLC patients after SBRT [15–21]. Our study is the first to describe an association with NLR and distant failure which contrasts with prior smaller studies [16, 18]. Additionally we are the first to show NLR is associated with poor PFS outcomes as a continuous variable and dichotomous variable with a cutoff of 3.28.

An association with PFS and distant failure suggests neutrophils could serve as a therapeutic target for intervention in high NLR patients to improve disease outcomes. Interventions that block TGFβ activity or enhance type I interferon activity at the tumor microenvironment could facilitate neutrophil anti-tumor cytotoxicity [34]. Emerging clinical data suggest radiation plays a key role in the reactivation of the anti-tumor immune response [35].

The immunomodulatory effect of single fraction SBRT on increasing intra-tumor and peripheral blood effector T cells has been shown in humans [36, 37]. Thus, following radiation effector T Cells flood into the tumor. Over the course of 4 weeks, these T-Cell are reduced and suppressor T-Cell numbers increase. Therefore, it is logical that another radiation fraction too close to the first fraction may wipe out the effector T-Cell population. Preclinical models also show a significant benefit to having a long period (10 days) between radiation treatments [38]. Comparison between 1 and 3 fraction SBRT regimens was not the purpose of this study, since such comparison has been already published [6]. However, as shown on Tables 2 and 3 fraction SBRT cohorts had a trend toward worse OS (*P* = 0.07) and PFS (*P* = 0.08). In current practice, the only lung SBRT regimen with such an interval between treatments is single fraction where the time to the next treatment is infinite. The use of single fraction SBRT in our cohort may thus explain why pre-treatment NLR was a statistically significant predictor of increased distant failure and poor PFS.

In ongoing clinical trials, PACIFIC-4 (NCT03833154) and SWOG 1914 (NCT04214262), adjuvant immunotherapy is being studied as a novel therapeutic to improve outcomes following SBRT in early-stage NSCLC. Possibly, immunotherapy may have the greatest benefit in patients at risk for poor outcomes with SBRT alone such as those with high NLR. Given these findings, consideration should be made for NLR to be tracked in SBRT trials.

### Limitations

Our study has limitations inherent in retrospective reviews including that some patients, especially those who came from a distance, had limited follow up. Too few local failures occurred in our patient cohort for analysis. Additionally, pre-treatment NLR data was collected at a single point in time, neglecting dynamic changes in NLR prior to or after treatment. Although treatment for adenocarcinoma and squamous cell NSCLC is different, separate cohorts for each histology were too small for any further analysis.

## Conclusions

In our single-institution study, NLR was an independent, adverse prognostic factor for poor distant recurrence, progression-free, and overall survival in peripheral early-stage NSCLC patients treated with single fraction SBRT.

## Supplementary Information

**Additional file 1:** **eTable 1. **Fine-Gray multivariate analysis for nodal and distant failure recurrences. **eTable 2. **Characteristics of NSCLC patients after propensity score matching (*n*=214). **eTable 3.** Logistic regression of NSCLC patient cohort to identify related variables to NLR.

## Data Availability

Data cannot be shared publicly because of protected health information. Data are available from the Institutional Data Access / Ethics Committee for researchers who meet the criteria for access to confidential data. Research data will be shared upon request to the corresponding author.
