# Supplementary material for: Association of neutrophil–lymphocyte ratio with survival in peripheral early-stage non-small cell lung cancer after stereotactic body radiation therapy
Source: BMC Cancer. 2023 Mar 18;23:254. doi: 10.1186/s12885-023-10719-3 (PMC10024425; doi:10.1186/s12885-023-10719-3)
Supplement: Supplementary file 1 — Additional file 1: eTable 1. Fine-Gray multivariate analysis for nodal and distant failure recurrences. eTable 2. Characteristics of NSCLC patients after propensity score matching (n=214). eTable 3. Logistic regression of NSCLC patient cohort to identify related variables to NLR. [file 12885_2023_10719_MOESM1_ESM.docx]

**eTable 1.** Fine-Gray multivariate analysis for nodal and distant failure recurrences.

|  | Nodal Failure | | Distant Failure | |
| --- | --- | --- | --- | --- |
|  | HR(95% CI) | P value | HR(95% CI) | P value |
| NLR | 1.08 (0.97-1.21) | 0.16 | 1.11 (1.05-1.18) | <0.001 |
| Age |  |  |  |  |
| <65 | 1 [Reference] | NA | 1 [Reference] | NA |
| ≥65 | 1.25 (0.33-4.71) | 0.75 | 0.89 (0.39-2.01) | 0.77 |
| Gender |  |  |  |  |
| Male | 1 [Reference] | NA | 1 [Reference] | NA |
| Female | 0.49 (0.22-1.13) | 0.1 | 0.74 (0.39-1.42) | 0.37 |
| Race |  |  |  |  |
| White | 1 [Reference] | NA | 1 [Reference] | NA |
| Other | 2.35 (0.44-12.70) | 0.32 | 2.90(0.79-10.60) | 0.11 |
| KPS |  |  |  |  |
| 70-100 | 1 [Reference] | NA | 1 [Reference] | NA |
| <70 | 0.59 (0.22-1.59) | 0.3 | 0.64 (0.31-1.31) | 0.22 |
| Histology |  |  |  |  |
| Adenocarcinoma | 1 [Reference] | NA | 1 [Reference] | NA |
| Squamous Cell | 1.57 (0.68-3.61) | 0.3 | 1.20 (0.65-2.22) | 0.55 |
| NSCLC (NOS) | 0.38 (0.08-1.79) | 0.22 | 0.21 (0.05-0.87) | 0.03 |
| Site |  |  |  |  |
| Left | 1 [Reference] | NA | 1 [Reference] | NA |
| Right | 0.90 (0.40-2.03) | 0.8 | 0.89 (0.47-1.70) | 0.72 |
| T staging |  |  |  |  |
| 1 | 1 [Reference] | NA | 1 [Reference] | NA |
| 2 | 1.57 (0.58-4.27) | 0.37 | 1.09 (0.51-2.34) | 0.83 |
| Fractions |  |  |  |  |
| 1 | 1 [Reference] | NA | 1 [Reference] | NA |
| 3 | 2.22 (0.94-5.23) | 0.07 | 2.11 (1.14-3.93) | 0.02 |
| Smoking Status |  |  |  |  |
| Current | 1 [Reference] | NA | 1 [Reference] | NA |
| Former | 0.53 (0.23-1.24) | 0.14 | 0.64 (0.34-1.21) | 0.17 |
| Never | 0.66 (0.06-6.83) | 0.73 | 0.65 (0.14-3.03) | 0.59 |
| Year of radiation |  |  |  |  |
| 2013 or earlier | 1 [Reference] | NA | 1 [Reference] | NA |
| 2013 or later | 0.49 (0.22-1.09) | 0.08 | 0.92 (0.47-1.80) | 0.81 |

aHR: adjusted hazards ratio; CI: confidence interval; KPS: Karnofsky performance; NOS: not otherwise specified.

**eTable 2.** Characteristics of NSCLC patients after propensity score matching (n=214).

|  | Low NLR (n=106) | High NLR (n=106) |  |
| --- | --- | --- | --- |
|  | Patients, No. (%) | | P value |
| Age |  |  |  |
| <65 | 15 (14.2) | 16 (15.1) | 1 |
| ≥65 | 91 (85.8) | 90 (84.9) |  |
| Gender |  |  |  |
| Male | 45 (42.5) | 43 (40.6) | 0.89 |
| Female | 61 (57.5) | 63 (59.4) |  |
| Race |  |  |  |
| White | 100 (94.3) | 101 (95.3) | 1 |
| Other | 6 (5.7) | 5 (4.7) |  |
| KPS |  |  |  |
| 70-100 | 92 (86.8) | 90 (84.9) | 0.67 |
| <70 | 14 (13.2) | 16 (15.1) |  |
| Histology |  |  |  |
| Adenocarcinoma | 59 (55.7) | 57 (53.8) | 0.96 |
| Squamous Cell | 36 (34.0) | 37 (34.9) |  |
| NSCLC (NOS) | 11 (10.4) | 12 (11.3) |  |
| Site |  |  |  |
| Left | 44 (41.5) | 51 (48.1) | 0.41 |
| Right | 62 (58.5) | 55 (51.9) |  |
| T staging |  |  |  |
| 1 | 88 (83.0) | 89 (84.0) | 1 |
| 2 | 18 (17.0) | 17 (16.0) |  |
| Fractions |  |  |  |
| 1 | 77 (72.6) | 76 (71.7) | 1 |
| 3 | 29 (27.4) | 30 (28.3) |  |
| Smoking Status |  |  |  |
| Current | 32 (30.2) | 29 (27.4) | 0.95 |
| Former | 67 (63.2) | 70 (66.0) |  |
| Never | 7 (6.6) | 7 (6.6) |  |
| Year of radiation |  |  |  |
| 2013 or earlier | 81 (76.4) | 82 (77.4) | 1 |
| 2013 or later | 25 (23.6) | 24 (22.6) |  |

KPS: Karnofsky performance; NOS: not otherwise specified.

**eTable 3.** Logistic regression of NSCLC patient cohort to identify related variables to NLR.

| Characteristic |  |  |
| --- | --- | --- |
|  | HR(95% CI) | P value |
| Age |  |  |
| <65 | 1 [Reference] | NA |
| ≥65 | 8.53 (0.43-1.68) | 0.64 |
| Gender |  |  |
| Male | 1 [Reference] | NA |
| Female | 0.72 (0.44-1.18) | 0.2 |
| Race |  |  |
| White | 1 [Reference] | NA |
| Other | 0.61 (0.18-1.86) | 0.4 |
| KPS |  |  |
| 70-100 | 1 [Reference] | NA |
| <70 | 1.29 (0.77-2.15) | 0.34 |
| Histology |  |  |
| Adenocarcinoma | 1 [Reference] | NA |
| Squamous Cell | 1.24 (0.72-2.14) | 0.43 |
| NSCLC (NOS) | 0.89 (0.40-1.95) | 0.77 |
| Site |  |  |
| Left | 1 [Reference] | NA |
| Right | 1.31 (0.80-2.16) | 0.28 |
| T staging |  |  |
| 1 | 1 [Reference] | NA |
| 2 | 1.70(0.88-3.32) | 0.12 |
| Fractions |  |  |
| 1 | 1 [Reference] | NA |
| 3 | 0.85 (0.47-1.54) | 0.6 |
| Smoking Status |  |  |
| Current | 1 [Reference] | NA |
| Former | 1.62 (0.54-1.17) | 0.09 |
| Never | 0.98 (0.32-2.87) | 0.97 |
| Year of radiation |  |  |
| 2013 or earlier | 1 [Reference] | NA |
| 2013 or later | 0.72 (0.38-1.33) | 0.29 |

aHR: adjusted hazards ratio; CI: confidence interval; KPS: Karnofsky performance; NOS: not otherwise specified.
